# Assessing the Quality and Impact of eHealth Tools: Systematic Literature Review and Narrative Synthesis

**DOI:** 10.2196/45143

**Published:** 2023-03-23

**Authors:** Christine Jacob, Johan Lindeque, Alexander Klein, Chris Ivory, Sabina Heuss, Marc K Peter

**Affiliations:** 1 FHNW - University of Applied Sciences Northwestern Switzerland Windisch Switzerland; 2 FHNW - University of Applied Sciences Northwestern Switzerland Olten Switzerland; 3 Medical Affairs (Personalised Healthcare and Patient Access), F Hoffmann-La Roche Ltd Basel Switzerland; 4 Innovation Management, Mälardalens University Västerås Sweden

**Keywords:** eHealth, mobile health, mHealth, digital health, technology assessment, technology adoption, technology implementation

## Abstract

**Background:**

Technological advancements have opened the path for many technology providers to easily develop and introduce eHealth tools to the public. The use of these tools is increasingly recognized as a critical quality driver in health care; however, choosing a quality tool from the myriad of tools available for a specific health need does not come without challenges.

**Objective:**

This review aimed to systematically investigate the literature to understand the different approaches and criteria used to assess the quality and impact of eHealth tools by considering sociotechnical factors (from technical, social, and organizational perspectives).

**Methods:**

A structured search was completed following the participants, intervention, comparators, and outcomes framework. We searched the PubMed, Cochrane, Web of Science, Scopus, and ProQuest databases for studies published between January 2012 and January 2022 in English, which yielded 675 results, of which 40 (5.9%) studies met the inclusion criteria. The PRISMA (Preferred Reporting Items for Systematic Reviews and Meta-Analyses) guidelines and the Cochrane Handbook for Systematic Reviews of Interventions were followed to ensure a systematic process. Extracted data were analyzed using NVivo (QSR International), with a thematic analysis and narrative synthesis of emergent themes.

**Results:**

Similar measures from the different papers, frameworks, and initiatives were aggregated into 36 unique criteria grouped into 13 clusters. Using the sociotechnical approach, we classified the relevant criteria into technical, social, and organizational assessment criteria. Technical assessment criteria were grouped into 5 clusters: technical aspects, functionality, content, data management, and design. Social assessment criteria were grouped into 4 clusters: human centricity, health outcomes, visible popularity metrics, and social aspects. Organizational assessment criteria were grouped into 4 clusters: sustainability and scalability, health care organization, health care context, and developer.

**Conclusions:**

This review builds on the growing body of research that investigates the criteria used to assess the quality and impact of eHealth tools and highlights the complexity and challenges facing these initiatives. It demonstrates that there is no single framework that is used uniformly to assess the quality and impact of eHealth tools. It also highlights the need for a more comprehensive approach that balances the social, organizational, and technical assessment criteria in a way that reflects the complexity and interdependence of the health care ecosystem and is aligned with the factors affecting users’ adoption to ensure uptake and adherence in the long term.

## Introduction

### Background

Research has shown that eHealth solutions may help optimize the quality of health care services [1-6] but also that the lack of a standardized assessment approach makes it challenging to select the appropriate tool for a particular purpose in a particular context [7-9]. eHealth tools continue to grow in number, creating a cluttered landscape that can be hard to navigate. Regarding mobile health apps alone, there are >300,000 available in the app stores, and >200 new apps are added daily [10]. Stakeholders, including patients, clinicians, payers, and other industry players such as pharmaceutical companies, face challenges in identifying quality in this crowded space [7,8]. It has also been established that users are faced with a situation where only a fraction of the available solutions are in fact appropriate for use [11], with considerable variation in the evidence supporting the different eHealth interventions [12]. Hence, there is a need for standardized assessment criteria to support informed decision-making with respect to eHealth tool evaluation [8].

Technological advancements have opened a path for many technology providers to easily develop and introduce eHealth tools to the public. The use of these tools is increasingly recognized as a critical quality driver in health care [13]; however, choosing a quality tool from the myriad of tools available for a specific health purpose is challenging. Moreover, rapid technological development means that many eHealth tools remain unevaluated by researchers [9,14], leaving potential users largely uninformed about their quality, veracity, safety, and fit [15]. Owing to this lack of proper assessment mechanisms, previous researchers that tried to assess existing apps have concluded that many eHealth tools that hit the market lack some relevant functionality and features [16] or do not fully satisfy users’ needs [17]. Furthermore, the crowded eHealth landscape compared with the number of approved prescription drugs, for instance, makes it quite challenging for both clinicians and patients to find, evaluate, and adopt the right eHealth tools [18]. Quite often, clinicians find themselves in a situation where they do not know which tool to use or recommend [19,20]. Failure to properly assess criteria such as the accuracy and appropriateness of eHealth tools can also compromise patient safety [21]. Ultimately, the lack of standardized and rigorous assessment frameworks results in tools that do not always meet high-quality standards across multiple domains [17].

### Objectives

The aim of this study was to build a better understanding of the different criteria used to assess the quality and impact of eHealth technologies. We adopted the World Health Organization (WHO) definition of eHealth as “the cost-effective and secure use of information and communications technologies in support of health and health-related fields, including health care services, health surveillance, health literature, and health education, knowledge and research” [22]. Furthermore, this review focused on patient-facing eHealth tools, including self-management tools and remote eHealth solutions, rather than tools used within and between care providers (eg, health care professional videoconferences or electronic health record integration) or health data analytics systems used at the population level.

Accordingly, a systematic review was conducted to provide a precise and up-to-date description of the different criteria used in published research to assess the quality and impact of eHealth tools from technological, social, and organizational perspectives. It also reflected on the potential implications and suggested directions for relevant stakeholders on how to best assess the eHealth tools that they are considering. This work builds on and expands the initial findings of a previous research project that investigated the sociotechnical factors affecting mobile health adoption from patients’ and clinicians’ perspectives, which have already been published [23,24].

Findings from this study will help inform clinicians, pharmaceutical executives, insurance professionals, technology providers, and policy makers by presenting them with an up-to-date and comprehensive review of the different criteria used to assess the quality and impact of eHealth tools as reported in the academic literature. This can guide them in making more informed decisions about which tools to use, endorse to patients, invest in, partner with, or reimburse based on their potential quality and impact.

## Methods

### Overview

The methods for this review were drawn from the PRISMA (Preferred Reporting Items for Systematic Reviews and Meta-Analyses) guidelines [25] and the Cochrane Handbook for Systematic Reviews of Interventions [26], both of which provide guidance toward a rigorous and reliable literature review methodology. The review methods were defined in advance, and the protocol was published in the Research Registry (reference: reviewregistry1291) and is available on the web to promote transparency [27]. This analysis did not require any major divergence from the initial protocol. The research question that guided this review was as follows: what are the technical, social, and organizational criteria that must be considered when assessing the quality and impact of eHealth tools?

### Search Strategy

A search of the PubMed, Cochrane, Web of Science, Scopus, and ProQuest databases in January 2022 identified relevant studies. The scope of this review was narrowed to studies published in English between January 2012 and January 2022. Only original, peer-reviewed, and published papers were included in this study. Other forms, such as editorials, unsystematic reviews, interviews, commentaries, unstructured observations, and position papers, were excluded. We decided not to include articles based on manual searches of reference lists in alignment with the guidance of the Cochrane Handbook for Systematic Reviews of Interventions that “positive studies are more likely to be cited” and “retrieving literature by scanning reference lists may thus produce a biased sample of studies” [26].

The search string shown in Textbox 1 was developed according to the participants, intervention, comparators, and outcomes framework. The authors limited the search of this search string to the manuscript title to make sure that the resulting papers were about eHealth assessment criteria as a whole, not individual assessments of pilot studies singling out specific tools. Comparators were not applicable to this study.

**The search string according to the participants, intervention, comparators, and outcomes framework.**

**Participants: patients**

- Focus on patient-facing eHealth technologies, including self-management tools and remote eHealth solutions, rather than tools used within and between care providers (eg, health care professional videoconferences or electronic health record integration) or health data analytics systems used at the population level

**Intervention: eHealth**

- “eHealth” OR “mobile health” OR “Telehealth” OR “mHealth” OR “mobile applications” OR “mobile apps” OR “telemonitoring” OR “app” OR “online health apps” OR “digital health” OR “health apps” OR “health platforms”

**Outcome: assessment criteria**

- AND (“assessment” OR “assess” OR “evaluation evaluating” OR “validation” OR “impact” OR “effectiveness” OR “efficacy” OR “quality”)
- AND (“criteria” OR “framework” OR “method” OR “methodology” OR “methodologies” OR “measurement” OR “toolkit” OR “tool” OR “tools” OR “approach” OR “scorecard” OR “path”)

### Study Selection

In total, 2 researchers (CJ and JL) were involved in the screening, eligibility, and inclusion phases, and any divergence was agreed upon through discussion between them. In cases where they could not reach an agreement, a third reviewer (SH for social or health-related criteria, CI for organizational criteria, and MP for technical criteria) discussed it with them and made the final decision. The practice partner (AK) ensured that the naming and categorization of the assessment criteria were relevant and meaningful from a practice point of view. The research team used the open-source app Rayyan (Qatar Computing Research Institute) to facilitate collaborative screening by the team [28]. Screening lasted from February 2022 to June 2022. The inclusion and exclusion criteria are detailed in Textbox 2 and were developed according to the participants, intervention, comparators, and outcomes framework.

After completing screening and resolving any conflicting views among the researchers, the selected full texts were assessed for eligibility independently by CJ and JL. Any disagreements were resolved through discussion with SH for social or health-related criteria, CI for organizational criteria, and MP for technical criteria. The risk of bias was assessed using the Critical Appraisal Skills Programme (CASP) checklist [29]. The checklist is provided in Multimedia Appendix 1, and it evaluates the following key quality criteria of the included studies: whether there was a clear statement of the aims of the research, whether the methodology was appropriate for the research objectives, whether the research design was appropriate to address the aims, whether the recruitment strategy was appropriate for the aims of the research, whether the data were collected in a way that addressed the research issue, whether the role of the researchers was adequately considered, whether ethical issues were considered, whether the data analysis was sufficiently rigorous, whether there was a clear statement of findings, and whether the researchers discussed the contribution the study made to existing knowledge or understanding (eg, did they consider the findings in relation to current practice or policy or relevant research-based literature). A Microsoft Excel (Microsoft Corp) sheet with the results of the appraisal of the included studies can be accessed in Multimedia Appendix 2 [15-21,30-62].

**Inclusion and exclusion criteria according to the participants, intervention, comparators, and outcomes framework.**

**Inclusion criteria**

- Participants
  Focused on patients
- Intervention
  Focused on patient-facing eHealth tools, including self-management tools and remote eHealth solutions
- Comparators
  Does not apply
- Outcomes
  Addresses the different criteria used to assess the quality and impact of eHealth tools regardless of the condition
- Publication type
  Original, peer-reviewed, and published papers
- Time frame
  Studies published between January 2012 and January 2022
- Language
  Studies published in English

**Exclusion criteria**

- Participants
  Focused solely on clinicians or technology providers
- Intervention
  Tools used within and between care providers (eg, health care professional videoconferences or electronic health record integration) or health data analytics systems used at the population level
- Comparators
  Does not apply
- Outcomes
  Individual assessments of pilot studies singling out specific tools
- Publication type
  Editorials, interviews, commentaries, unstructured observations, and position papers
- Time frame
  Studies published before January 2012 or after January 2022
- Language
  Studies published in other languages

### Data Collection and Synthesis

The variety of procedures and results that were identified in the included studies was not homogeneous enough to enable a quantitative analysis of the data. Therefore, a narrative synthesis was used and structured around the social, organizational, and technical criteria used to assess the quality and impact of eHealth tools. NVivo (QSR International), a computer-assisted qualitative data analysis software, was used to assist with this task.

Data coding began with a preliminary data extraction grid that included themes based on previous research and technology acceptance frameworks; the initial codebook was informed by our previous work that aggregated the factors affecting adoption from patients’ and clinicians’ perspectives [23,24,63]. More codes were added as they emerged during the review process. Thematic analysis by Braun and Clarke [64-66] was used to identify and extract themes under the social, technical, and organizational assessment criteria addressed in the research question. Social criteria included any social-related elements, such as the effects of people and groups influencing one another through culture; technical criteria included elements related to the material sides of the technology, such as its ease of use and usability; and organizational criteria were linked to elements such as resources and workflow. The phases of the thematic analysis are explained in detail in Multimedia Appendix 3. The 7 key phases were data familiarization; initial code generation; searching for themes; reviewing themes; defining and naming themes; linking themes to explanatory frameworks; and, finally, producing the report. This process lasted from June 2022 to September 2022.

### Theoretical Framework

Health care technologies are generally more complex than tools that address a specific user need. They typically serve patients with comorbidities who are mostly treated by multidisciplinary teams of clinicians potentially working across more than one organization. This particular nature of the health care sector calls for a wider view that goes beyond a tool’s technical aspects as health care technology cannot be successfully implemented in isolation from the broader context in which it is being used [63].

Therefore, the authors were guided in their thinking by the sociotechnical theory, which has at its core the idea that the design and performance of any innovation can only be understood and improved if both “social” and “technical” aspects are brought together and treated as interdependent parts of a complex system [67]. In social studies of technology and, more specifically, the sociotechnical theory, technology, roles, and practices and organizational structures are viewed as interacting parts of mutually interdependent collections of elements [67]. This position is aligned with what several scholars have recommended (explaining that many of the broadly used frameworks adopt a technology-centered view focusing on the technological aspects [68,69]): a shift to multidimensional models that go past technology to encompass the surrounding context as well as societal and implementation factors [68-71]. Therefore, the resulting criteria go beyond the technical quality of eHealth tools to also cover all other relevant aspects, such as social and organizational criteria.

## Results

### Study Selection Flow and Characteristics of the Included Studies

**Figure 1:**
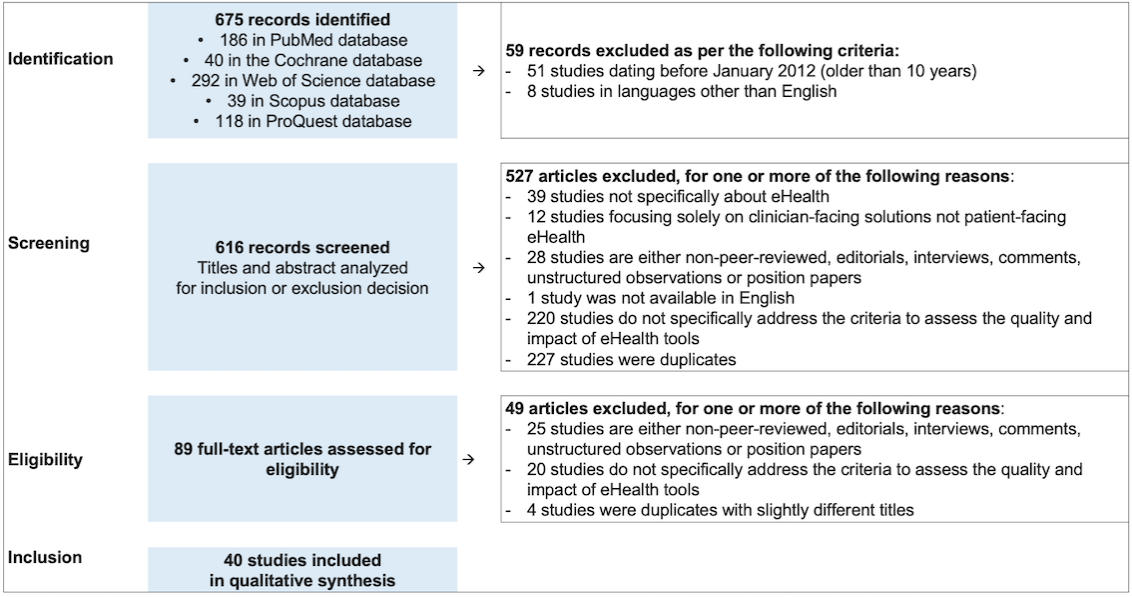
Study selection flow diagram based on the PRISMA (Preferred Reporting Items for Systematic Reviews and Meta-Analyses) guidelines.

The PRISMA study selection flow diagram in Figure 1 depicts the flow of information through the different phases of the systematic review. It maps out the number of records identified, included, and excluded and the reasons for exclusion. This process resulted in the inclusion of 40 articles for the qualitative synthesis [15-21,30-62]. Multimedia Appendix 4 [15-21,30-62] presents the sample characteristics of the included studies from research methodology, geographical, and clinical focus perspectives.

### Critical Appraisal

We assessed the quality of the included studies using the CASP checklist for qualitative studies [29]. We chose the CASP because of the diversity of methodologies used in the included studies and the narrative nature of our own synthesis (as opposed to meta-analysis and more quantitative methodologies) and because it is the most commonly used tool for quality appraisal in health-related qualitative evidence synthesis, with endorsement from the Cochrane Qualitative and Implementation Methods Group [72]. The included studies encompassed diverse methodologies, including quantitative, qualitative, and mixed methods as well as systematic literature reviews; hence, some of the questions on the checklist were not applicable to all types of studies. Scores were not assigned as this was not recommended by the checklist [29].

On the basis of the critical appraisal, of the 40 studies, 4 (10%) did not clearly justify their choice of study design but still used a design that was suitable for their objectives, 3 (8%) did not provide sufficient details on the profiles of the assessors and implications for potential bias, 5 (12%) did not report whether the study procedure was reviewed for ethics approval or how they protected the privacy of the participants, 12 (30%) were not clear enough about their data analysis strategy and whether it was sufficiently rigorous, and 4 (10%) did not sufficiently discuss the practical or policy implications of their findings. The quality assessment results are provided in Multimedia Appendix 2.

Studies were not excluded based on quality assessment outcome as this was unlikely to have a major influence on the definition of the assessment criteria and the resulting aggregated framework. However, the assessment provided a general idea of the quality of the development processes of the existing frameworks and, therefore, the strength of the evidence [73]. This will be discussed in more detail in the *Discussion* section when addressing the challenges with existing initiatives and frameworks.

### Frameworks and Guidelines That Resulted From or Were Used in the Included Studies

**Figure 2:**
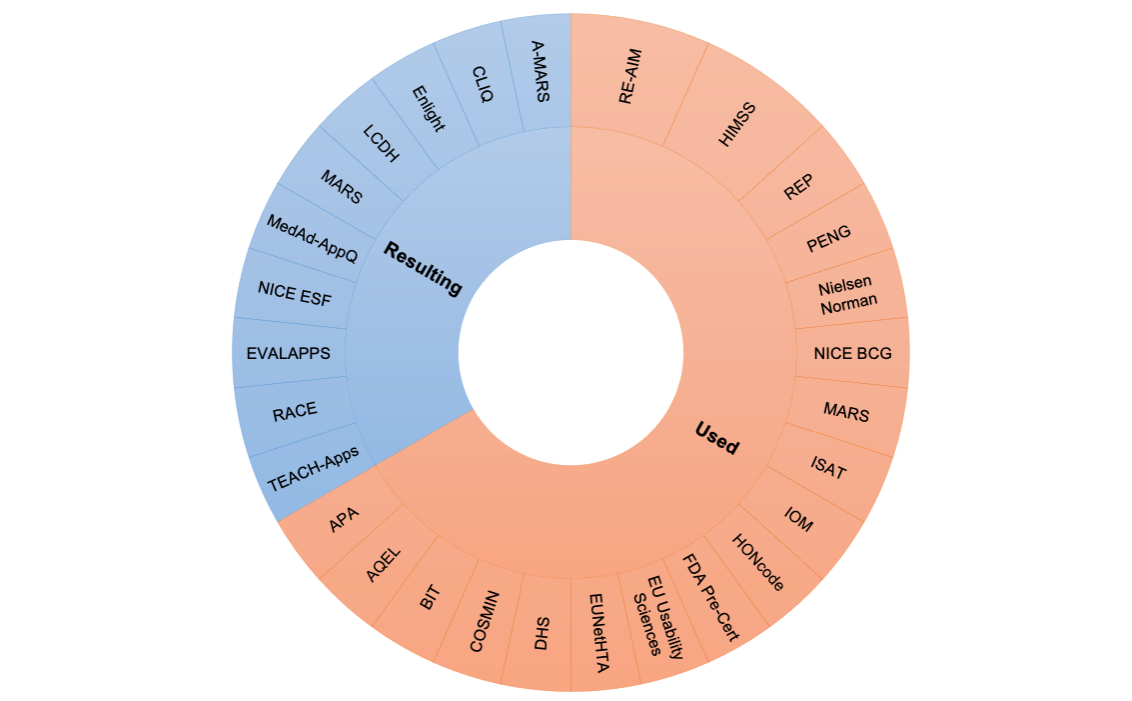
Frameworks and guidelines used in or resulting from the included studies according to their occurrence. A-MARS: adapted Mobile App Rating Scale; APA: American Psychiatric Association app evaluation framework; AQEL: App Quality Evaluation framework; BIT: Behavior Interventions Using Technology framework; CLIQ: Clinical Information Quality framework; COSMIN: Consensus-Based Standards for the Selection of Health Measurement Instruments; DHS: Digital Health Scorecard; EU: European Union; EUNetHTA: European Network for Health Technology Assessment Core Model; EVALAPPS: an app assessment instrument in the field of overweight and obesity management; FDA Pre-Cert: Food and Drug Administration precertification program; HIMSS: Health Care Information and Management Systems Society criteria framework; HONcode: Health On the Net foundation code of conduct; IMO: quality improvement framework of the Institute of Medicine; ISAT: Intervention Scalability Assessment Tool; LCDH: Legal Challenges in Digital Health framework; MARS: Mobile App Rating Scale; MedAd-AppQ: Medication Adherence App Quality assessment tool; NICE BCG: National Institute for Health and Care Excellence behavior change guidance; NICE ESF: National Institute for Health and Care Excellence Evidence Standards Framework for digital health and care technologies; PENG: Swedish acronym that stands for “Prioritering efter NyttoGrunder,” translated to “Prioritizing based on contribution of benefits”; RACE: Review, Assess, Classify, and Evaluate; RE-AIM: reach, effectiveness, adoption, implementation, and maintenance framework; REP: Replicating Effective Programs; TEACH-apps: Technology Evaluation and Assessment Criteria for Health Apps.

Several publications (21/40, 52%) did not mention the use of a framework; however, there were 19 different frameworks or guidelines used, and 22% (9/40) of the studies resulted in the creation of a new assessment framework. Figure 2 presents the diversity of the frameworks used in or resulting from the included studies according to their occurrence. A framework resulting from a study means that this framework was the end result of the work in that study, whereas a framework used in a study was the starting point rather than the outcome of that study.

Stoyanov et al [55] created the Mobile App Rating Scale (MARS), and Roberts et al [21] adapted it, creating the adapted MARS (A-MARS) to make it appropriate for the evaluation of both mobile phone apps and e-tools, whereas EVALAPPS was the outcome of the work by Robles et al [62]. The Clinical Information Quality (CLIQ) framework for digital health resulted from the work by Fadahunsi et al [37], whereas the work by Baumel et al [32] resulted in the creation of Enlight, a comprehensive quality and therapeutic potential evaluation tool for mobile and web-based eHealth interventions.

Garell et al [38] focused on evaluating digital health services according to current legislation by creating a framework for assessing the legal challenges in developing digital health services, the Legal Challenges in Digital Health (LCDH) framework, whereas the Medication Adherence App Quality (MedAd-AppQ) assessment tool resulted from the work by Ali et al [16]. The updated National Institute for Health and Care Excellence Evidence Standards Framework (NICE ESF) for digital health and care technologies was the result of the work by Unsworth et al [56], whereas Varshney et al [57] created the Review, Assess, Classify, and Evaluate (RACE) process, and Camacho et al [18] created the Technology Evaluation and Assessment Criteria for Health Apps (TEACH-apps) process.

Of the frameworks and guidelines that were used in the included studies, only 2 were used twice, and the rest were only used once. The Health Care Information and Management Systems Society criteria framework [74,75] was used by Stoyanov et al [55] and Wildenbos et al [61]. The reach, effectiveness, adoption, implementation, and maintenance (RE-AIM) framework [76] was used by Blackman et al [34] and de La Vega et al [35], whereas the American Psychiatric Association (APA) app evaluation framework [77] was used by Camacho et al [18]. The App Quality Evaluation (AQEL) framework [78] was used by DiFilippo et al [36], and the Behavior Interventions Using Technology (BIT) framework [79] was used by de La Vega et al [35].

The Consensus-Based Standards for the Selection of Health Measurement Instruments initiative [80,81] was used by Muro-Culebras et al [50], whereas the Digital Health Scorecard [8,82] was used by Sedhom et al [17], and the European Network for Health Technology Assessment (EUNetHTA) Core Model [83,84] was used by von Huben et al [60]. Stoyanov et al [55] used the European Union UsabilityNet [85] and the Nielsen Norman user experience criteria [86]. The Food and Drug Administration (FDA) precertification program [87,88] was used by Alon et al [15], whereas Ali et al [16] used a version of the Health On the Net Foundation code of conduct [89,90] that was adapted to assess the reliability and credibility of medical apps [91,92]. The quality improvement framework of the Institute of Medicine (IOM) [93] was used by Lee et al [46].

The Intervention Scalability Assessment Tool (ISAT) [94] was used by Azevedo et al [30], whereas the National Institute for Health and Care Excellence behavior change guidance (NICE BCG) [95] was used by McMillan et al [48], and the *Prioritering efter NyttoGrunder* (PENG; translated as “Prioritizing based on contribution of benefits”) evaluation tool [96] was used by Parv et al [52]. Finally, the Replicating Effective Programs (REP) framework [97] was used by Camacho et al [18]. Multimedia Appendix 5 [8,15-18,21,30,32,34-38,46,48,50,52, 55-57,60-62,74-93,95-97] presents the frameworks and guidelines that resulted from or were used in the included studies and provides more details on their contexts and the assessment criteria that each of them encompassed.

### Synthesized Assessment Criteria

We synthesized similar measures from the different papers, frameworks, and initiatives, resulting in 36 unique criteria that mirrored all the relevant assessment methods that were cited in the included papers. It is worth noting that some of the criteria may fit into more than one category but were placed in the best-fitting category because of their importance and impact. For example, inclusive design could be considered a design aspect and could have been included in the design cluster under the technical assessment criteria; however, given its importance for human centricity and its social implications for health care equity, it was placed in that cluster instead. We also deliberately included assessment criteria that apply to high-risk eHealth tools as it allowed us to identify a more extensive list of criteria with the expectation that not all criteria will necessarily apply to lower-risk eHealth tools. For instance, the patient safety assessment criteria mostly apply for high-risk tools and would be less relevant for low-risk tools that do not endanger patient safety.

**Figure 3:**
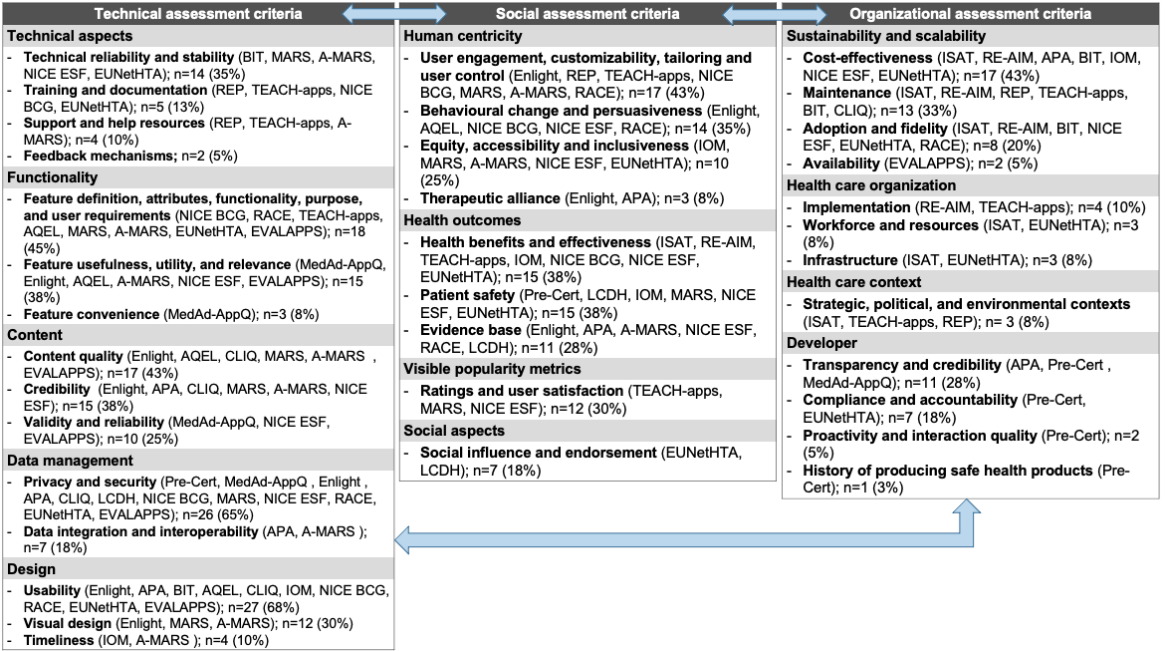
Aggregated assessment criteria, the frameworks that mentioned them, and their occurrence in the included studies. A-MARS: adapted Mobile App Rating Scale; APA: American Psychiatric Association app evaluation framework; AQEL: App Quality Evaluation framework; BIT: Behavior Interventions Using Technology framework; CLIQ: Clinical Information Quality framework; DHS: Digital Health Scorecard; EUNetHTA: European Network for Health Technology Assessment Core Model; FDA Pre-Cert: Food and Drug Administration precertification program; EVALAPPS: an app assessment instrument in the field of overweight and obesity management; ISAT: Intervention Scalability Assessment Tool; LCDH: Legal Challenges in Digital Health framework; MARS: Mobile App Rating Scale; MedAd-AppQ: Medication Adherence App Quality assessment tool; NICE BCG: National Institute for Health and Care Excellence behavior change guidance; NICE ESF: National Institute for Health and Care Excellence Evidence Standards Framework for digital health and care technologies; RACE: Review, Assess, Classify, and Evaluate; RE-AIM: reach, effectiveness, adoption, implementation, and maintenance framework; REP: Replicating Effective Programs; TEACH-apps: Technology Evaluation and Assessment Criteria for Health Apps.

Using sociotechnical theory as a guide, we classified the relevant criteria into technical, social, and organizational criteria, as detailed in Figure 3, which shows the aggregated criteria from all the included studies, the frameworks that mentioned each criteria listed in brackets, and their occurrence. The double-ended arrows in the figure signal the interplay between the technical, social, and organizational aspects. For instance, the social criteria related to human centricity and inclusive design would also affect and be affected by the technical criteria related to the tool’s design, such as usability. Similarly, the health care organization organizational criteria, such as infrastructure and implementation, will affect and be affected by the technical criteria related to data integration and interoperability. Multimedia Appendix 6 [15-21,30-62] reflects the assessment criteria classified according to the sociotechnical approach, the respective frameworks where they prevailed, their occurrences in the included studies, their definitions, and the respective references.

### Technical Assessment Criteria

The technical assessment criteria were grouped into 5 clusters: technical aspects, functionality, content, data management, and design. The technical aspects cluster includes technical reliability and stability (BIT, MARS, A-MARS, NICE ESF, and EUNetHTA; 14/40, 35%), which typically refer to the system quality of the tool from a technical perspective and potential technical issues (eg, errors, freezing, and response time of the application); training and documentation (REP, TEACH-apps, NICE BCG, and EUNetHTA; 5/40, 12%), such as the availability of material and assistance for end users to ensure their comfort with basic competencies and skills needed to use the tool effectively (eg, in the form of training material, videos, or documentation); support and help resources (REP, TEACH-apps, and A-MARS; 4/40, 10%), usually referring to the ease with which help or support can be accessed via the tool; and feedback mechanisms (2/40, 5%), meaning the possibility to provide instant feedback through the tool (eg, provider messaging).

The functionality cluster includes feature definition, attributes, functionality, purpose, and user requirements (NICE BCG, RACE, TEACH-apps, AQEL, MARS, A-MARS, EUNetHTA, and EVALAPPS; 18/40, 45%), defined as the presence of well-defined features, purpose clarity and expected use, what symptoms or health issues are addressed, and whether the features match end-user requirements; feature usefulness, utility, and relevance (MedAd-AppQ, Enlight, AQEL, A-MARS, NICE ESF, and EVALAPPS; 15/40, 38%), meaning appropriate and relevant features to meet the clinical aim, the right mix of ability and motivation, and meeting the intended purpose; and feature convenience (MedAd-AppQ; 3/40, 8%), which typically assesses how convenient or bothersome some of the features are, such as reminders, push notifications, and daily prompts.

The content cluster includes content quality (Enlight, AQEL, CLIQ, MARS, A-MARS, and EVALAPPS; 17/40, 42%), which assesses the quality of the health-related content (accuracy, completeness, consistency, and timeliness); content credibility (Enlight, APA, CLIQ, MARS, A-MARS, and NICE ESF; 15/40, 38%), which looks into content source credibility (eg, the WHO), advisory support, third-party verification, or the level of clinicians’ involvement in the tool’s content development; and content validity and reliability (MedAd-AppQ, NICE ESF, and EVALAPPS; 10/40, 25%), typically defined as the extent to which a tool’s contents are relevant to the underlying construct and likely to be effective in achieving a particular intervention purpose in a specific intended population.

The data management cluster includes data privacy and security (FDA precertification program [Pre-Cert], MedAd-AppQ, Enlight, APA, CLIQ, LCDH, NICE BCG, MARS, NICE ESF, RACE, EUNetHTA, and EVALAPPS; 26/40, 65%)—which assess the cybersecurity responsibility, presence of disclaimers, informed consent, and privacy policy and whether the treatment of any data is compatible with the Patient Data Act, Personal Data Act, and other applicable privacy laws—and data integration and interoperability (APA and A-MARS; 7/40, 18%), which evaluate the tool’s ability to exchange information with and use information from other health technologies (eg, electronic health records) and users’ ability to smoothly move across different platforms.

The design cluster includes the tool’s usability (Enlight, APA, BIT, AQEL, CLIQ, IOM, NICE BCG, RACE, EUNetHTA, and EVALAPPS; 27/40, 68%), which assesses user experience, navigation, learnability, and ease of use; visual design (Enlight, MARS, and A-MARS; 12/40, 30%), which evaluates esthetics, layout, size, pop-up windows and flash images, visual appeal, and consistency of the theme throughout the tool; and timeliness (IOM and A-MARS; 4/40, 10%), typically defined as the ability to use the tool in real time (ie, real-time data tracking), reducing waits and sometimes harmful delays for both those who receive and those who provide care.

### Social Assessment Criteria

The social assessment criteria were grouped into 4 clusters: human centricity, health outcomes, visible popularity metrics, and social aspects. The human centricity cluster includes user engagement, customizability, tailoring, and user control (Enlight, REP, TEACH-apps, NICE BCG, MARS, A-MARS, and RACE; 17/40, 42%), meaning the tool’s interactivity and the ability to enable customization, collaboration, participation, information sharing, and decision-making in one’s own health as well as evidence for collaboration with users; behavior change and persuasiveness (Enlight, AQEL, NICE BCG, NICE ESF, and RACE; 14/40, 35%), which assess whether the tool reflects a persuasive design that aims to understand what influences people’s behavior and decision-making and then uses this information to design compelling user interactions (call for action, load reduction of activities, therapeutic rationale and pathway, rewards, real data-driven and adaptive, and ongoing feedback); equity, accessibility, and inclusiveness (IOM, MARS, A-MARS, NICE ESF, and EUNetHTA; 10/40, 25%), which look into whether the tool supports providing care that takes the user context into account and does not vary in quality because of personal characteristics such as gender, ethnicity, geographic location, and socioeconomic status (eg, tools that are accessible to vulnerable populations such as people with disabilities, patients with chronic diseases, patients with mental illnesses, pediatric patients, maternity patients, and older adults); and therapeutic alliance (Enlight and APA; 3/40, 8%), defined as the tool’s ability to foster interaction between clinicians and their patients.

The health outcomes cluster includes health benefits and effectiveness (ISAT, RE-AIM, TEACH-apps, IOM, NICE BCG, NICE ESF, and EUNetHTA; 15/40, 38%), which typically assess evidence of effectiveness of the new technology in producing health benefits in a real-world setting, also referred to as real-world evidence; patient safety (Pre-Cert, LCDH, IOM, MARS, NICE ESF, and EUNetHTA; 15/40, 38%), which looks into the ability of an eHealth tool to handle “dangerous” information entered by a patient and avoid safety risks to patients from the care that is intended to help them; and evidence base (Enlight, APA, A-MARS, NICE ESF, RACE, and LCDH; 11/40, 28%), which reflects the presence of solid scientific evidence supporting the tool’s health claims (eg, published research and randomized controlled trials).

The visible popularity metrics cluster includes ratings and user satisfaction (TEACH-apps, MARS, and NICE ESF; 12/40, 30%), which reflect users’ perceived value through users’ reviews and ratings (as a proxy for quality, usefulness, or acceptability and popularity). Finally, the social aspects cluster includes social influence and endorsement (EUNetHTA and LCDH; 7/40, 18%), which assess the possibilities for peer support, social networking, information sharing, and endorsement by health care professionals.

### Organizational Assessment Criteria

The organizational assessment criteria were grouped into 4 clusters: sustainability and scalability, health care organization, health care context, and developer. The sustainability and scalability cluster includes cost-effectiveness (ISAT, RE-AIM, APA, BIT, IOM, NICE ESF, and EUNetHTA; 17/40, 42%), which evaluates the balance between the costs and benefits arising from the tool’s use. This refers to the tool’s direct costs (eg, purchase price, subscription, and licensing) but may also include costs associated with the tool’s selection, staff training, setting up support mechanisms, and appropriate governance. This cluster also includes maintenance (ISAT, RE-AIM, REP, TEACH-apps, BIT, and CLIQ; 13/40, 32%), which assesses the commitment of the developers to maintaining their products in the long term by conducting periodic updates and maintenance (from both technical and content perspectives); adoption and fidelity (ISAT, RE-AIM, BIT, NICE ESF, EUNetHTA, and RACE; 8/40, 20%), which look into the tool’s adoption rates, acceptability, and desirability as well as its integration into clinical practice, system use, and adherence; and availability (EVALAPPS; 2/40, 5%), which evaluates the guarantee of access to the tool and its data at any time and its availability on different operating systems (eg, Android and iOS).

The health care organization cluster includes implementation (RE-AIM and TEACH-apps; 4/40, 10%), which assesses the extent to which the intervention was delivered as intended (eg, feasibility of delivering all components of an intervention at a predetermined date and time); workforce and resources (ISAT and EUNetHTA; 3/40, 8%), which assess the workforce required to scale up the tool and the implications for care processes and care management; and infrastructure (ISAT and EUNetHTA; 3/40, 8%), which assesses the readiness of the necessary infrastructure for the tool’s implementation. The health care context cluster includes strategic, political, and environmental contexts (ISAT, TEACH-apps, and REP; 3/40, 8%) and evaluates how favorable are the preconditions (strategic, political, and environmental contexts) that influence the scaling up of the eHealth tool, for example, the intervention’s suitability to the socioeconomic context in question, considerations of foreign languages that the tool needs to support, literacy level, and the local regulatory environment.

The developer cluster includes the transparency and credibility of the tool’s developer (APA, Pre-Cert, and MedAd-AppQ; 11/40, 28%), which look into the availability of information and credentials of the individuals and organizations involved in the development and funding of the tool; compliance and accountability (Pre-Cert and EUNetHTA; 7/40, 18%), which assess the developer’s ethical conduct, clinical responsibility, and respect for the rules and regulations protecting patients’ rights and societal interests; proactivity and interaction quality (Pre-Cert; 2/40, 5%), which evaluate the interaction quality between the provider and the users, including responsiveness, after-sales services, and customer orientation as well as the demonstration of excellence in a proactive approach to the assessment of user needs and continuous learning; and, finally, the history of producing safe health products (Pre-Cert; 1/40, 2%), which assesses whether the developer has successfully delivered safe health products in the past.

## Discussion

### A Scattered and Fragmented Landscape

**Figure 4:**
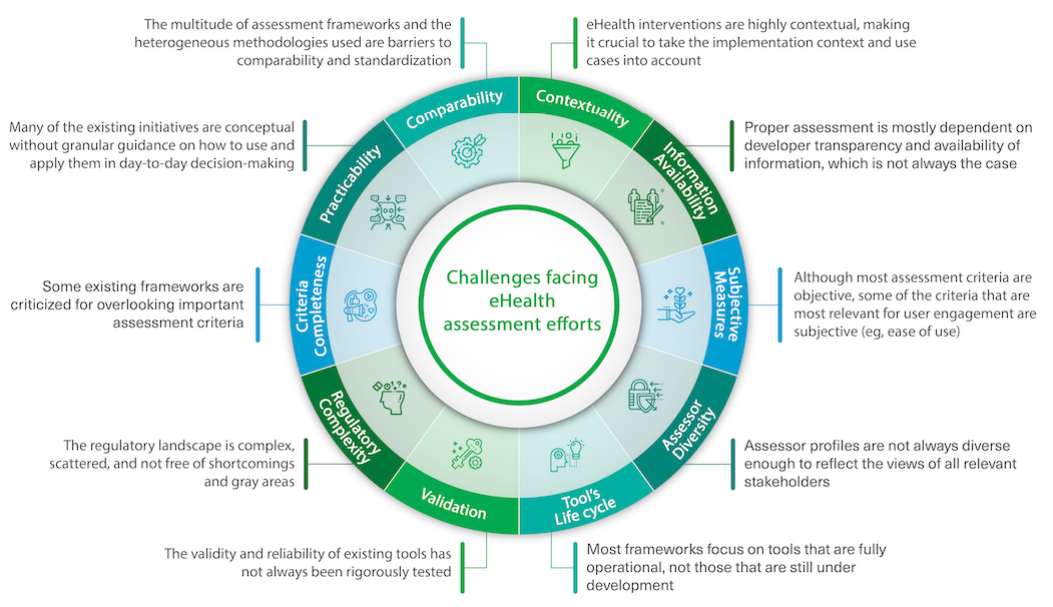
Challenges facing eHealth assessment efforts.

Although there are various initiatives working on finding ways to assess the quality of eHealth tools, these efforts face multiple challenges, as shown in the overview in Figure 4.

#### Comparability

The multitude of frameworks and initiatives attempting to address the topic of eHealth tool assessment shows the lack of standardization in this field and adds another challenge for the relevant stakeholders as they are faced with proliferating approaches and not knowing which assessment tool to use or how best to use it [98,99]. The diversity of assessment methods sometimes results in a lack of clarity or comparability [20,30,32,35,36,39]; furthermore, this scattered landscape also signals the lack of generalizability and standardization in this field of research [32]. Moreover, assessment and data collection methods vary widely between the different initiatives (eg, self-reported vs objective measures and qualitative vs quantitative assessment) [34,37,39,50,98-100].

#### Practicability

In many cases, there is limited information and methods describing how to realistically assess and evaluate these tools in practice [19,33]; many of the existing initiatives are conceptual without granular guidance on how to use and apply them in day-to-day decision-making [37,56,59,82]. For instance, the work by Kloc et al [101] compared the English NICE ESF for digital health technologies and the French National Authority for Health guide on the assessment of connected medical device guidelines and concluded that the guidelines do not always clearly describe the assessment process or the specific criteria determining the decision. Correspondingly, Bradway et al [99] suggested that users should be provided with guidance and educational resources on how to perform a proper assessment.

#### Criteria Completeness

Moreover, research has shown that some of the existing initiatives sometimes overlook important assessment criteria, resulting in incomplete or issue-specific assessment formworks [32,35,36,51,99].

#### Regulatory Complexity

The lack of regulatory clarity and the absence of institutionalized quality controls in many countries make a comprehensive definition of the assessment criteria more challenging [15,41-43,53]. Moreover, there are some shortcomings with some of the current certification labels, as highlighted by Bradway et al [99], who pointed out that, even though common labels may categorize a tool as a medical device, it may still include the warning in fine print that it is intended for entertainment only, showing a lack of accountability and creating confusion on the users’ side. There are also many gray areas in existing regulatory oversight efforts; for instance, the US FDA applies regulatory oversight only to a small subset of tools that qualify as medical devices and potentially pose a risk to patient safety [9,102]. The European regulatory system offers another model in which each member state can file an approval application for a high-risk medical device and obtain a Conformité Européenne mark. However, although Conformité Européenne marks indicate that these tools are compliant with European legislation, the tools only need to demonstrate safety and performance but not clinical efficacy [102]. These regulatory gaps mean that the safety, efficacy, and ethical compliance of certified eHealth tools cannot be guaranteed, posing a potential threat to patients’ safety [103].

#### Validation

Furthermore, the validity and reliability of the existing assessment tools and frameworks have not always been rigorously tested [17,50,56]; such validation efforts are key to ensure assessment processes that reﬂect the real-world needs of the different stakeholders in the health care ecosystem [17].

#### Contextuality

Relatedly, eHealth interventions are highly contextual, making it crucial to consider the implementation context and use cases, but the varying contexts and use cases make it quite challenging to find a standardized and generalizable way to assess them [15,17,18,100].

#### Information Availability

Proper assessment is mostly dependent on developer transparency and the availability of information, which is unfortunately not always the case, making it quite challenging to address the questions needed to accurately assess the quality and impact of an eHealth tool [9,98]. Concerningly, a previous study showed that, in a sample of 52 eHealth tools, 63.5% of the providers gave no information about the tool itself, 67.3% did not provide information about the credentials of the developers or consultants, and only 4% provided information supporting the tool’s efficacy [104].

#### Subjective Measures

Although most assessment criteria are objective, some of the criteria that are most relevant for user engagement are subjective, as pointed out by Lagan et al [98], limiting the standardization of the assessment outcome. For example, given the importance of user engagement for the success of eHealth tools [23,24,105,106], it would still be crucial to include assessment criteria that reflect key user engagement and adoption drivers such as ease of use and visual appeal [23,24,63,107].

#### Assessor Diversity

In addition, as Bradway et al [99] noted, some assessment initiatives do not involve or even inform all the relevant stakeholders of assessment results, establishing the importance of involving diverse assessor profiles, including the tools’ developers themselves.

#### Tool’s Life Cycle

Finally, most existing assessment frameworks focus only on eHealth tools that are fully operational within the market and do not necessarily tackle those that are still under development or have not been implemented yet [99]. One of the few assessment frameworks that look into specific criteria for the different phases of the development and implementation cycle is the framework for the design and evaluation of digital health interventions developed by Kowatsch et al [108] categorizing the assessment criteria according to the phase in which the tool is in terms of preparation, optimization, evaluation, and implementation.

It is worth noting that most national initiatives are also still in their infancy and facing several teething problems, which shows that these frameworks have not reached a high enough maturity level yet. For instance, even though Germany became the first country worldwide to approve certain eHealth tools, referred to as *Digitale Gesundheitsanwendungen* (DiGA) in German, meaning digital health applications, for prescription with costs covered by standard statutory health insurance, research has shown that clinicians’ adoption rates of this option are still rather low [109]. Similarly, the FDA has recently announced that its Pre-Cert program, which focuses on medical technology providers and their internal processes rather than on individual devices and apps, is still not ready to go beyond the pilot phase [110,111]. In addition, Alon et al [15] stated that they were unable to identify a standard measure that differentiated the tools requiring regulatory review from those that did not when they assessed the Pre-Cert program.

Despite these challenges, efforts to harmonize and standardize assessment approaches are ongoing. For example, the European Committee for Standardization (CEN) International Organization for Standardization (ISO) technical specification for the quality and reliability of health and wellness apps (CEN ISO/TS 82304-2), published in 2021, provides quality requirements for health apps and defines a health app quality label to visualize the quality and reliability of these apps [112]. Horizon Europe project “Label2Enable” involves 14 organizations from 7 countries (Belgium, Croatia, Germany, Italy, Lithuania, the Netherlands, and Spain) that have joined forces to promote the CEN ISO/TS 82304-2 health app assessment framework and label in Europe [113].

### The Relevance of a Sociotechnical Approach to eHealth Assessment

Despite the multitude of initiatives attempting to address this topic, it remains that there are multiple challenges to be addressed. It is also clear that developing a comprehensive assessment criteria framework for eHealth will be challenging owing to its multidimensional nature [19,41-43]. The findings from this systematic review show that there is no single framework that is used uniformly to evaluate the different assessment criteria of eHealth tools. However, it is worth noting that, despite their different contexts and the different disease conditions they addressed, there was substantial overlap among the frameworks. Nevertheless, although these initiatives attempt to provide relevant information on the quality of eHealth tools, they are not always able to address all stakeholder issues, and although most criteria can be related to one framework or another, no framework seems to cover all relevant criteria without being extended.

**Figure 5:**
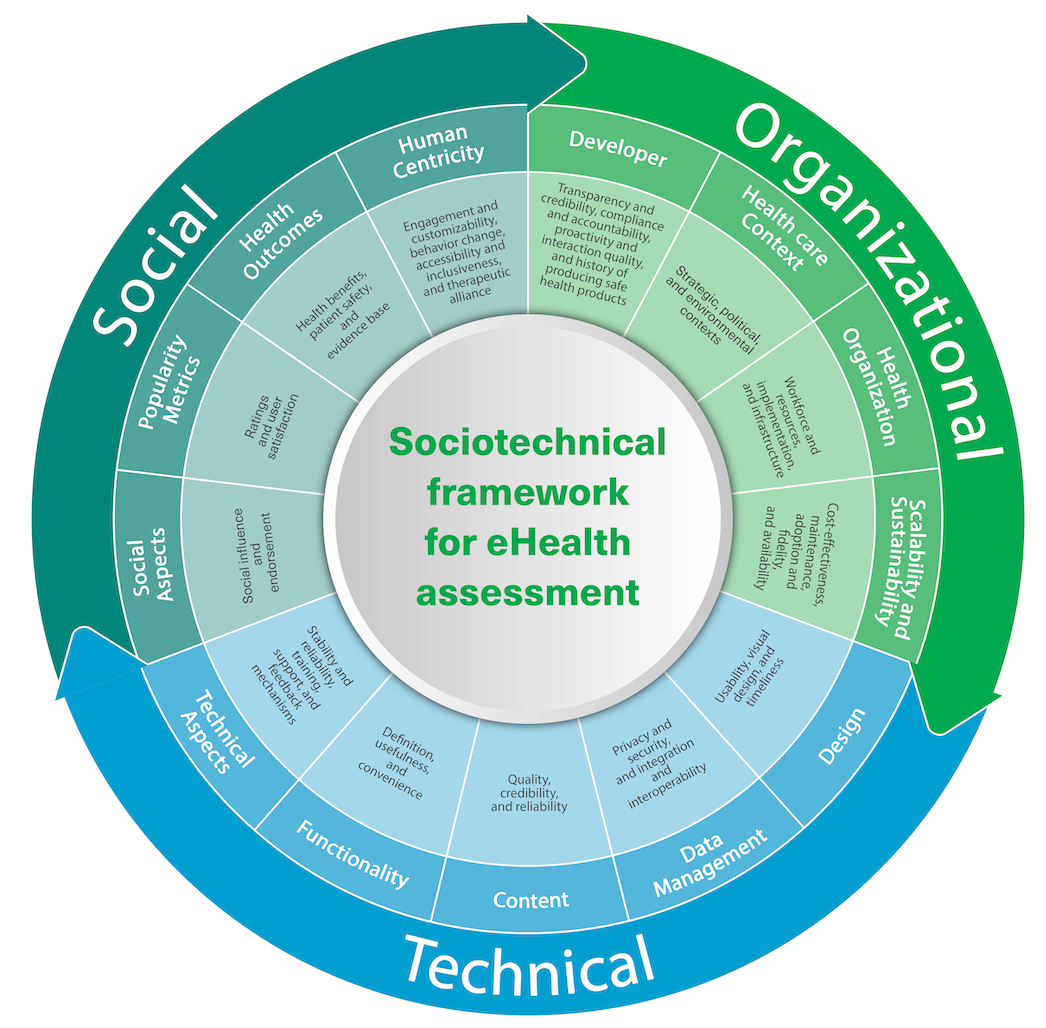
Sociotechnical framework to assess the quality and impact of eHealth tools.

We propose an aggregated framework adopting a sociotechnical approach to eHealth evaluation balancing the technical, social, and organizational assessment criteria. This aggregated framework considers all the criteria appearing in the included studies and classifies them according to the sociotechnical framework; this aggregation should help overcome some of the identified challenges with current efforts, namely, incomplete assessment measures [114]. Our approach also acknowledges that health care technology cannot be successfully implemented and scaled in isolation from the broader organizational and social contexts in which it is being used and that, therefore, we need to use frameworks that consider implementation challenges in light of the complexity of the sociotechnical structure and interplay between the technical, social, and organizational aspects. Figure 5 summarizes our proposed aggregated framework that considers all the criteria covered in the included studies, classifying them according to the sociotechnical framework. The arrows in the figure indicate the continuity and interconnectedness between the social, organizational, and technical criteria.

Technical assessment criteria are the foundation for the viability of any eHealth solution and for it to be considered at all by potential users; without this foundation, a tool would not properly meet the basic requirements for success. This is most likely why technical aspects have mostly been the focus of existing initiatives and frameworks [51]. For instance, the only assessment criteria that were reflected in more than half of the included studies were the tools’ usability (27/40, 68%) and data privacy and security (26/40, 65%), highlighting the current focus on assessing the technical aspects without necessarily giving enough weight to social and organizational assessment measures, as demonstrated in our previous discussion. This was similarly highlighted by Lagan et al [98], who pointed out the rising popularity of data privacy criteria in assessment frameworks in recent years.

Ensuring a high level of technical performance and offering well-defined and useful functionalities and features as well as credible, valid, and reliable content; proper data management strategies; and a superior user experience are the basics that every eHealth tool must meet for it to be considered by the relevant users. Even though feature usefulness may seem like an intuitive and basic requirement for the success of any eHealth technology, Singh et al [54] reported that their evaluation of 143 tools targeting patients who have high needs and incur high health care costs showed that only a minority of these tools appeared likely to be useful to patients.

It is also worth noting that, although data integration and interoperability were only mentioned in 18% (7/40) of the included studies, previous studies have shown that this is an important user requirement. User adoption research has shown that interoperability issues can raise clear concerns when eHealth tools cannot be integrated into the hospital’s or clinic’s current systems or when there are limitations in data integration and exchange [23,63]. This technical criterion closely affects and is affected by the organizational criteria related to infrastructure and implementation. It is also closely related to the sustainability and scalability organizational criteria, showing the interconnectedness between these elements that contribute to the potential success of a given eHealth tool.

The inclusion of organizational assessment criteria may help address a key challenge with current efforts related to the importance of the contextuality of eHealth tools as these technologies are not used in isolation of the health care ecosystem; therefore, a proper assessment of the potential impact of these tools should consider the specific context. Health care technologies are generally more complex than tools that address individual user needs as they usually support patients with comorbidities who are typically treated by multidisciplinary teams that might even work in different health care organizations, hence the importance of contextual and organizational aspects to assess the potential impact of these novel solutions. Context-specific criteria such as implementation, workforce and resources, infrastructure, and the overall health care context do not seem to be fairly represented in the current assessment initiatives. Our analysis showed that only 10% (4/40) of the included studies encompassed implementation criteria, and only 8% (3/40) looked into the required infrastructure, workforce, and resources as well as social, political, and environmental contexts. This results in situations where a tool may be of good quality when assessed in isolation but might not have the desired impact in a real-life scenario because of contextual criteria that do not necessarily allow it to be successfully implemented or scaled if not properly evaluated.

To put things into perspective, it is important to consider the factors affecting user adoption when assessing potential eHealth tools to avoid situations where a tool may be of good quality in isolation of its context but not a good fit when rolled out in a real-life setting. A comprehensive systematic review that looked into the factors affecting clinician adoption of eHealth tools in 171 published studies indicated that organizational factors, especially workflow-related factors such as implications for the workload and workflow, the infrastructure required for the implementation, and the wider health care context such as local regulations, are crucial for clinician adoption [23,63], showing some disconnect between the focus of the current assessment efforts and what it takes for a tool to be successfully adopted by its intended users in a real-life context.

Even though the availability of information is one of the challenges facing current initiatives, as explained in the previous section, less than one-third (11/40, 28%) of the included studies incorporated organizational assessment criteria regarding the developers’ transparency and credibility. Our approach proposes the inclusion of developer-related criteria by evaluating the developers’ transparency and credibility, compliance and accountability, proactivity and interaction quality, and history of producing safe tools to help overcome this challenge and entice tool providers to transparently communicate the information needed for their very own assessment.

Hence, the overall organizational assessment criteria should comprise criteria regarding the sustainability and scalability of the tool (cost-effectiveness, maintenance, adoption and fidelity, and availability); criteria related to health care organizations in the specific context being assessed (implementation, workforce and resources, and infrastructure); criteria related to the wider health care context, such as local regulations and certification requirements; and criteria to assess the developers’ credibility, compliance, and interaction quality.

We equally advocate for the importance of the inclusion of relevant social assessment criteria that evaluate the potential societal impact of these tools. Notably, even though many frameworks included usability in general as an assessment criterion, more than half (23/40, 58%) of the included studies did not specifically address human centricity through active user engagement and behavior change strategies. This is concerning considering the lack of reliable evidence regarding the ability of most commercially available eHealth tools to induce lasting behavior change [99,115]. Proper user engagement and effective behavior change design strategies may help address issues reported in previous studies that established that only a small fraction of patients kept using eHealth tools in the long term and that up to 80% of users would only show minimal engagement, using the tools <2 times [116,117]. Another study conducted on a large real-world cohort of 189,770 people reported that only 2.6% of the people who downloaded an eHealth tool sustained its active use [118], concluding that the impact of such tools may remain minimal if they fail to properly engage patients, making this a vital assessment criterion. Although developers seem to pay less attention to behavior maintenance than to initiation and evidence for collaboration with users or professionals is mostly lacking, as reported by McMillan et al [48], promisingly, Baumel et al [31] noted some advancements made in recent years as human-centric criteria related to persuasive design and therapeutic alliance gain more importance. This social criterion closely affects and is affected by the technical criteria related to a tool’s design and usability.

Nonetheless, 75% (30/40) of the included studies failed to address some core social principles, such as the equity, accessibility, and inclusiveness of the tools being assessed, overlooking the vital societal impact of such criteria. We highlight the importance of the inclusion of these measures as inclusive design principles may help developers address the needs of the most susceptible patient populations who may not be engaging with such technologies owing to their age, health-related physical and cognitive challenges, educational level, socioeconomic status, or technological skills and experience [24]. Designing for inclusivity does not ignore the unique features, environments, and cultural contexts of users; many aspects of the digital divide may be addressed through an inclusive design that incorporates cultural appropriateness, easy-to-understand lay language that does not require high literacy levels, and ease of use that does not require any sophisticated technical skills [24]. Unfortunately, equity seems to be one of the less frequently observed criteria in eHealth tools, as equally reported by Lee et al [46] and confirmed by our findings. Assessing such criteria would increase the chances of having tools that are designed in a way that makes them more accessible to the very patients who need them the most.

Surprisingly, less than 40% (15/40, 38%) of the included studies considered criteria related to health outcomes, such as health benefits and effectiveness, patient safety, and evidence base. This may affect the societal impact of these tools if not assessed when determining a specific tool’s potential impact on health, which is supposed to be the main reason why people use these tools, especially when previous studies have indicated that the clinical benefit of many of these tools is quite limited or insufficient, as reported by Huckvale et al [91]. This social criterion is closely affected by the technical criteria related to a tool’s features and content.

Generally, comprehensive social assessment criteria according to our findings should encompass human centricity (by assessing user engagement, customizability, behavior change strategies, the tool’s inclusiveness, and its impact on the therapeutic alliance), health outcomes (by assessing health benefits and effectiveness, patient safety, and evidence base), visible popularity metrics such as tool ratings and user satisfaction, and other influential aspects such as social influence and endorsement.

### Limitations and Future Research

This study contributes to the understanding of the different criteria used to assess the quality and impact of eHealth tools; however, some limitations must be acknowledged. This review may not have included relevant studies that were not indexed in the searched databases or were written in a language other than English as well as gray literature searches that could have also allowed for the identification of additional relevant insights. However, this study focused on peer-reviewed scientific papers. In addition, this analysis only considered published studies, and no further contact was made with the authors of the papers to obtain additional information or validate our thematic analysis. We also did not include articles based on manual searches of reference lists to avoid a biased sample of studies given that positive studies are more likely to be cited. Consequently, it is possible that other frameworks, initiatives, or assessment criteria were missed.

Future work could include studies in other languages to gain a better grasp of any interregional or intercultural differences. The authors also intend to build on this review by conducting another study to critically apply, reflect, validate, and revise the criteria aggregated in this study with the relevant stakeholders and cocreate accessible and easy-to-use tools with practice experts that may support them in their eHealth assessment decisions.

### Conclusions

The findings from this systematic review demonstrate that there is no single framework that is used uniformly to assess the quality and impact of eHealth tools. Current assessment efforts face some core challenges, such as the lack of comparability and practicability, gaps in criteria completeness of the individual frameworks, regulatory complexity, issues with the validation of existing frameworks, the contextuality of eHealth tools, the availability of the information necessary for the assessment, the need to include subjective measures, and the lack of assessor diversity in many cases. This review also highlights the need for a more comprehensive approach that balances the social, organizational, and technical assessment criteria in a way that reflects the complexity and interdependence of the health care ecosystem and is aligned with the factors affecting users’ adoption to ensure uptake and adherence in the long term.

Our proposed framework aggregates and expands the criteria appearing in the included studies and classifies them according to the sociotechnical framework, acknowledging that health care technologies cannot be successfully implemented and scaled in isolation from the broader organizational and social contexts in which they are being used and that, therefore, we need to use frameworks that consider implementation challenges in light of the complexity of the sociotechnical structure and interplay between the technical, social, and organizational aspects. More efforts are needed to find ways to overcome the identified challenges and validate the aggregated framework resulting from this study with the relevant stakeholders to ensure its pertinence and help make it more usable and accessible to potential assessors to support a more comprehensive process of evaluating the quality and impact of eHealth technologies.

## Appendix

Multimedia Appendix 1 Critical Appraisal Skills Programme checklist.

Multimedia Appendix 2 Critical appraisal of the included studies.

Multimedia Appendix 3 Phases of thematic analysis based on the work by Braun and Clarke [31-33].

Multimedia Appendix 4 Characteristics of the included studies.

Multimedia Appendix 5 Frameworks and guidelines that resulted from or were used in the included studies.

Multimedia Appendix 6 Assessment criteria and their occurrence, with references.

## Abbreviations

A-MARS: adapted Mobile App Rating Scale
APA: American Psychiatric Association
AQEL: App Quality Evaluation
BIT: Behavior Interventions Using Technology
CASP: Critical Appraisal Skills Programme
CEN: European Committee for Standardization
CLIQ: Clinical Information Quality
DiGA: Digitale Gesundheitsanwendungen
EUNetHTA: European Network for Health Technology Assessment
FDA: Food and Drug Administration
IOM: Institute of Medicine
ISAT: Intervention Scalability Assessment Tool
ISO: International Organization for Standardization
LCDH: Legal Challenges in Digital Health
MARS: Mobile App Rating Scale
MedAd-AppQ: Medication Adherence App Quality
NICE BCG: National Institute for Health and Care Excellence behavior change guidance
NICE ESF: National Institute for Health and Care Excellence Evidence Standards Framework
PENG: Prioritering efter NyttoGrunder
Pre-Cert: Food and Drug Administration precertification program
PRISMA: Preferred Reporting Items for Systematic Reviews and Meta-Analyses
RACE: Review, Assess, Classify, and Evaluate
RE-AIM: reach, effectiveness, adoption, implementation, and maintenance
REP: Replicating Effective Programs
TEACH-apps: Technology Evaluation and Assessment Criteria for Health Apps
WHO: World Health Organization
