# Supplementary material for: Assessing the Quality and Impact of eHealth Tools: Systematic Literature Review and Narrative Synthesis
Source: JMIR Hum Factors. 2023 Mar 23;10:e45143. doi: 10.2196/45143 (PMC10131913; doi:10.2196/45143)
Supplement: Multimedia Appendix 4 [file humanfactors_v10i1e45143_app4.pdf]

Appendix 4. Characteristics of included studies.

Study characteristic (n)		References
**Study design**		
	Mixed methods (12)	[15,16,20,32,42,47,52,56–58,61,62]
	Quantitative (7)	[17,21,36,43,45,51,55]
	Qualitative (6)	[18,38,46,48,53,54]
	Randomized control trial (RCT) (2)	[35,49]
	Systematic review (13)	[19,30,31,33,34,37,39–41,44,50,59,60]
**Disease area or focus**		
	Mental health (3)	[20,21,55]
	Health behavior change (3)	[40,48,49]
	Pediatrics (2)	[35,53]
	Adolescents (1)	[44]
	Asthma (1)	[42]
	Diabetes (1)	[52]
	Hypertension (1)	[36]
	Medication adherence (1)	[16]
	Noncommunicable diseases (1)	[60]
	Obesity (1)	[62]
	Oncology (1)	[17]
	Opioid use (1)	[57]
	Physical activity (1)	[34]
	Self-tracking (1)	[43]
	Telemonitoring (1)	[30]
	Non-specific (20)	[15,18,19,31–33,37–39,41,45–47,50,51,54,56,58,59,61]
**Location**		
	The United States (5)	[15,18,32,35,36]
	Sweden (2)	[38,49]
	The United Kingdom (2)	[48,56]
	Australia (1)	[21]
	Canada (1)	[20]
	Estonia (1)	[52]
	Iran (1)	[51]
	Korea (1)	[46]
	Portugal (1)	[30]
	Singapore (1)	[16]

	Spain (1)	[62]
	Non-specific (22)	[17,19,31,33,34,37,39–41,43–45,47,50,53–55,57–61]
