# Supplementary material for: Assessing the Quality and Impact of eHealth Tools: Systematic Literature Review and Narrative Synthesis
Source: JMIR Hum Factors. 2023 Mar 23;10:e45143. doi: 10.2196/45143 (PMC10131913; doi:10.2196/45143)
Supplement: Multimedia Appendix 5 [file humanfactors_v10i1e45143_app5.pdf]

Appendix 5: Frameworks and guidelines that resulted from or were used in the included studies

Framework	Reference(s) where it occurred in the included studies	Reference(s) where the framework originated	Assessment criteria
A-MARS: Adapted Mobile App Rating Scale	Roberts et al [21]	Roberts et al [21]	Adapted the original MARS to make it appropriate for the evaluation of both mobile phone applications as well as e-tools. Its evaluation criteria encompass engagement, functionality, aesthetics, information, subjective quality, and health-related measures.
APA: American Psychiatric Association App Evaluation framework	Camacho et al [18]	Torous et al [77]	Offers a simple and ethically grounded approach that first considers access, privacy, safety, evidence, usability, and clinical integration.
AQEL: App Quality Evaluation framework	Di Filippo et al [36]	Di Filippo et al [78]	Evaluates behavior change potential, knowledge support, skill development potential, app functionality, and meeting the intended purpose, plus 2 modifiable domains: (modification-specified) app appropriateness for target age group, and relevance to the target audience.
BIT: Behavior Interventions using Technology framework	De La Vega [35]	Hermes et al [79]	Assesses acceptability, adoption, appropriateness, feasibility, fidelity, implementation cost, penetration, and sustainability.
CLIQ: Clinical Information Quality framework	Fadahunsi et al [37]	Fadahunsi et al [37]	Includes 13 unique dimensions: accessibility, completeness, portability, security, timeliness, accuracy, interpretability, plausibility, provenance, relevance, conformance, consistency, and maintainability, which are categorized into 3 clusters: availability, informativeness, and usability.
COSMIN: Consensus-based Standards for the Selection of Health Measurement Instruments	Muro-Culebras et al [50]	Terwee et al, Prinsen et al [80,81]	Aims to standardize the psychometric criteria necessary to validate patient-reported outcome measures. The 10 psychometric characteristics described in COSMIN are internal consistency, reliability, measurement error, content validity, structural validity, hypotheses testing, cross-cultural validity, criterion validity, responsiveness, and interpretability.
DHS: the Digital Health Scorecard	Sedhom et al [17]	Mathews et al [8,82]	Was developed by John Hopkins researchers, and assesses a tool's technical performance, clinical outcomes, usability, and cost.
Enlight	Baumel et al [32]	Baumel et al [32]	Enlight's rating measures are divided into quality assessments and checklists. Quality assessments include usability, visual design, user engagement, content, therapeutic persuasiveness, therapeutic alliance, and general subjective evaluation; and the checklists include credibility, privacy, security, and evidence-base.
EUNetHTA: the Health Technology Assessments Core Model	von Huben et al [60]	EUnetHTA joint action, O'Rourke et al [83,84]	Assesses new technologies against existing health care interventions based on comparative safety, clinical, and cost-effectiveness
EU UsabilityNet	Stoyanov et al [55]	UsabilityNet [85]	A project funded by the European Union to provide resources and networking for usability practitioners.

EVALAPPS: an app assessment instrument in the field of overweight and obesity management	Robles et al [62]	Robles et al [62]	Assesses the efficacy, effectiveness, and safety of mobile health applications.
FDA Pre-Cert: the food and drug administration pre-certification program	Alon et al [15]	US food and drug administration [87,88]	Aims to speed up the review of new medical apps, the program focuses on assessing whether the developer meets 5 key criteria: patient safety, product quality, clinical responsibility, cybersecurity responsibility, and proactive culture.
HIMSS: Health Information and Management Systems Society criteria framework	Stoyanov et al [55], Wildenbos et al [61]	HIMSS, Xcertia [74,75]	Was published as early as 2012 and updated in 2019 under the name Xcertia Guidelines. This guidance assesses an app's privacy, security, operability, usability, and content.
HONcode: Health on the Net foundation code of conduct	Ali et al [16]	Boyer et al, Ranasinghe et al [89,90]<br>Huckvale et al, Lewis et al [91,92]	A version of the Health on the Net foundation code of conduct (HONcode) that was adapted to assess the reliability and credibility of medical apps.
IOM: quality improvement framework of the Institute of Medicine	Lee et al [46]	Institute of Medicine [93]	The six aims for quality improvement focus on safety, effectiveness, equity, timeliness, patient-centeredness, and efficiency.
ISAT: Intervention Scalability Assessment Tool	Azevedo et al [30]	Milat et al [94]	Considers the context in which the intervention is being considered for scale-up and consists of five domains: the problem, the intervention, strategic and political contexts, evidence of effectiveness, and intervention costs and benefits. And assesses the potential implementation and scale-up requirements within five domains: fidelity and adaptation, reach and acceptability, delivery setting and workforce, implementation infrastructure, and sustainability.
LCDH: Legal Challenges in Digital Health Framework	Garell et al [38]	Garell et al [38]	The framework assesses whether an eHealth tool is considered a medical device, and looks into criteria such as medical responsibility, care damage, data management, and consent.
MARS: Mobile App Rating Scale	Stoyanov et al. [55], Roberts et al [21]	Stoyanov et al. [55]	A widely cited assessment scale that uses 4 overarching dimensions; engagement, functionality, aesthetics, and information
MedAd-AppQ: Medication Adherence App Quality assessment tool	Ali et al [16]	Ali et al [16]	Focuses on assessing three key sections: content reliability, feature usefulness, and feature convenience
NICE BCG: the NICE behavior change guidance	McMillan et al [48]	National Institute for Health and Care Excellence [95]	Covers a set of principles that can be used to help people change their behavior by assessing criteria such as the tool's purpose, usability, tailoring, behavior change technique, maintenance, documentation, and data protection.
NICE ESF: NICE Evidence Standards Framework for digital health and care technologies	Unsworth et al [56]	Unsworth et al [56]	Designed to provide a standardized approach to guide the relevant stakeholders on the levels of evidence needed for the clinical and economic evaluation of eHealth tools, and segregates the evidence requirements according to the risk level of the tool being assessed. The ESF clusters tools into three tiers, from lowest evidence requirement to highest: tier A: system impact, tier B: understanding and communicating, and tier C: interventions.
Nielsen Norman	Stoyanov et al [55]	Nielsen Norman Group [86]	The Nielsen Norman Group's user experience (UX) criteria.

PENG: Swedish acronym which stands for “Prioritering efter NyttoGrunder”, translated to “Prioritizing based on contribution of benefits”	Parv et al [52]	Rowe et al [96]	Focuses on mapping and quantifying the costs and benefits arising from eHealth tools. It is a structural method of evaluating all the different types of benefits that Information and Communication Technology (ICT) generates within a project, where tangible and intangible benefits are evaluated in monetary units.
RACE: Review, Assess, Classify, and Evaluate	Varsheny et al [57]	Varsheny et al [57]	Focuses on studying m-health apps and their specific application for opioids. It uses a 3-step approach in a successive manner to identify and analyze eHealth tools. Main evaluation criteria are purposeful design, functionality and usability, ethical considerations, reach and impact, public health and clinical guidelines.
RE-AIM: reach, effectiveness, adoption, implementation, and maintenance framework	Blackman et al [34], De La Vega et al [35]	Glasgow et al [76]	Assesses 5 dimensions: reach, efficacy, adoption, implementation, and maintenance.
REP: Replicating Effective Programs	Camacho et al [18]	Kilbourne et al [97]	Consists of four phases: pre-conditions (e.g., identifying need, target population, and suitable intervention), pre-implementation (e.g., intervention packaging and community input), implementation (e.g., package dissemination, training, technical assistance, and evaluation), and maintenance and evolution (e.g., preparing the intervention for sustainability).
TEACH-Apps: Technology Evaluation and Assessment Criteria for Health Apps	Camacho et al [18]	Camacho et al [18]	Focuses on supporting users in selecting culturally appropriate and clinically relevant eHealth tools. The process comprises four parts: preconditions (e.g., gathering apps and considering local needs), pre-implementation (e.g., customizing criteria and offering digital skills training), implementation (e.g., evaluating apps and creating educational handouts), and maintenance and evolution (e.g., repeating the process every 90 days and updating content).
