# Supplementary material for: Assessing the Quality and Impact of eHealth Tools: Systematic Literature Review and Narrative Synthesis
Source: JMIR Hum Factors. 2023 Mar 23;10:e45143. doi: 10.2196/45143 (PMC10131913; doi:10.2196/45143)
Supplement: Multimedia Appendix 6 [file humanfactors_v10i1e45143_app6.pdf]

Appendix 4: Assessment criteria classified according to the sociotechnical approach, the respective frameworks where they prevailed, their occurrences in the included studies, their definitions, and references. A-MARS: Adapted Mobile App Rating Scale; APA: American Psychiatric Association App Evaluation framework; AQEL: App Quality Evaluation framework; BIT: Behavior Interventions using Technology framework; CLIQ: Clinical Information Quality framework; DHS: the Digital Health Scorecard; EUNetHTA: the Health Technology Assessments Core Model; FDA Pre-Cert: the food and drug administration pre-certification program; EVALAPPS: an app assessment instrument in the field of overweight and obesity management; ISAT: Intervention Scalability Assessment Tool; LCDH: Legal Challenges in Digital Health Framework; MARS: Mobile App Rating Scale; MedAd-AppQ: Medication Adherence App Quality assessment tool; NICE BCG: the NICE behavior change guidance; NICE ESF: NICE Evidence Standards Framework for digital health and care technologies; RACE: Review, Assess, Classify, and Evaluate; RE-AIM: reach, effectiveness, adoption, implementation, and maintenance framework; REP: Replicating Effective Programs; TEACH-Apps: Technology Evaluation and Assessment Criteria for Health Apps

	Criteria	Framework(s)	Occurrence n (%)	Definition as per the included studies	Studies where the criteria emerged
**1. Technical assessment criteria**					
**1.a. Technical aspects**					
	Technical reliability and stability	BIT - MARS - A-MARS - NICE ESF - EUNetHTA	14 (35%)	System quality, reliability and stability of the tool from a technical perspective, potential technical issues (e.g., errors, unexpected stops of running, response time of the application)	[17,19–21,35,41,42,44,47,51,55,56,59,60]
	Training and documentation	REP - TEACH-apps - NICE BCG - EUNetHTA	5 (13%)	Assistance for end users to ensure their comfort with basic competencies and skills needed to use the tool effectively (e.g., in the form of training material, videos, or documentation)	[18,19,48,59,60]
	Support and help resources	REP - TEACH-apps - A-MARS	4 (10%)	The ease with which help or support can be accessed via the tool	[17–19,21]
	Feedback mechanisms	--	2 (5%)	The possibility to give instant feedback through the tool (e.g., provider messaging)	[17,53]
**1.b. Functionality**					
	Feature definition, attributes, functionality, purpose, and user requirements	NICE BCG - RACE - TEACH-apps - AQEL - MARS - A-MARS – EUNetHTA - EVALAPPS	18 (45%)	The presence of well-defined features, purpose clarity and expected utilization, what symptoms or health issues are addressed, and whether the features match end-user requirements	[15,17–21,36,39,41,42,44,48,50,55,57,59,60,62]
	Feature usefulness, utility, and relevance	MedAd-AppQ - Enlight - AQEL - A-MARS - NICE ESF - EVALAPPS	15 (38%)	Appropriate and relevant features to meet clinical aim, right mix of ability and motivation, and meeting the intended purpose	[16,17,20,21,32,36,41,42,44,49,50,54,56,60,62]

	Feature convenience	MedAd-AppQ	3 (8%)	How convenient or bothersome are some of the features such as reminders, push notifications, and daily prompts	[16,41,49]
**1.c. Content**					
	Content quality	Enlight - AQEL - CLIQ - MARS - A-MARS- EVALAPPS	17 (43%)	Quality of the health-related content (accuracy, completeness, consistency, timeliness)	[19,21,31–33,36,37,39,41,42,44,47,49,50,55,58,62]
	Credibility	Enlight - APA - CLIQ - MARS - A-MARS - NICE ESF	15 (38%)	Content source credibility (e.g., WHO), advisory support, third party verification, level of healthcare professional involvement in the tool's content development	[17–19,21,31–33,37,39,44,50,53,55,56,58]
	Validity and reliability	MedAd-AppQ - NICE ESF- EVALAPPS	10 (25%)	The extent to which a tool's contents are relevant to the underlying construct and likely to be effective in achieving a particular intervention purpose in a specific intended population	[16,19,37,45,47,53,54,56,60,62]
**1.d. Data Management**					
	Privacy and security	Pre-Cert - MedAd-AppQ - Enlight - APA - CLIQ - LCDH - NICE BCG - MARS - NICE ESF - RACE – EUNetHTA - EVALAPPS	26 (65%)	Cybersecurity responsibility, the presence of disclaimers, informed consent, and privacy policy; and the treatment of any personal data is compatible with the Patient Data Act, Personal Data Act, and other applicable privacy laws	[15–20,31,32,37–39,41–44,47,48,51,53–57,59,60,62]
	Data integration and interoperability	APA - A-MARS	7 (18%)	The ability to exchange information with and use information from other health technologies (e.g., electronic health records), and users' ability to move across different platforms	[17,18,20,21,41,59,60]
**1.e. Design**					
	Usability	Enlight - APA - BIT - AQEL- CLIQ - IOM - NICE BCG - RACE - EUNetHTA- EVALAPPS	27 (68%)	User experience, navigation, learnability, and ease of use	[17–21,31–33,35–37,39,41,43–51,54,57,60–62]
	Visual design	Enlight - MARS - A-MARS	12 (30%)	Aesthetics, layout, size, popup windows and flash images, visual appeal, consistency of the theme throughout the tool	[17,19,21,31,32,35,41,44,47,50,55,61]
	Timeliness	IOM - A-MARS	4 (10%)	The ability to use the tool in real time (i.e. real-time data tracking), reducing waits and	[21,37,46,51]

				sometimes harmful delays for both those who receive and those who give care	
**2. Social assessment criteria**					
**2.a. Human centricity**					
	User engagement, customizability, tailoring, user control	Enlight - REP - TEACH-apps - NICE BCG - MARS -A-MARS - RACE	17 (43%)	Interactivity and the ability to enable customization, collaboration, participation, information-sharing, and decision-making in one's own health. Evidence for collaboration with users	[15,18–21,31,32,39,41–44,48,50,54,55,57]
	Behavioral change, persuasiveness	Enlight - AQEL- NICE BCG - NICE ESF - RACE	14 (35%)	A persuasive design that aims at understanding what influences people's behavior and decision making, and then uses this information to design compelling user interactions. Therapeutic persuasiveness (call for action, load reduction of activities, therapeutic rationale and pathway, rewards, real data driven/adaptive, ongoing feedback)	[19,31,32,36,40,42,44,45,48,49,51,56,57,60]
	Equity, accessibility, inclusiveness	IOM - MARS - A-MARS - NICE ESF - EUNetHTA	10 (25%)	Providing care that takes the user context into account and does not vary in quality because of personal characteristics, such as gender, ethnicity, geographic location, and socioeconomic status (e.g., tools that are accessible to vulnerable populations; the disabled, patients with chronic diseases, patients with mental diseases, pediatric patients, maternity patients, and the elderly)	[20,21,46,51,54–56,59–61]
	Therapeutic alliance	Enlight - APA	3 (8%)	The ability to foster the interaction between the health care professionals and their patients	[18,31,32]
**2.b. Health outcomes**					
	Health benefits and effectiveness	ISAT-RE-AIM - TEACH-apps - IOM - NICE BCG - NICE ESF - EUNetHTA	15 (38%)	Evidence of effectiveness of the new technology in producing health benefits in a real-world setting (RWE)	[18,20,21,30,34,35,39,45,46,48,51,52,56,59,60]
	Patient safety	Pre-Cert - LCDH - IOM - MARS - NICE ESF - EUNetHTA	15 (38%)	Avoiding injuries to patients from the care that is intended to help them, and the ability for an eHealth tool to handle "dangerous" information entered by a patient. Tool classification, and risk level (e.g., if it's a software as a medical was it certified or not)	[15,19,20,37,38,41,42,44,46,51,54–56,59,60]

	Evidence base	Enlight - APA - A-MARS - NICE ESF - RACE - LCDH	11 (28%)	Solid scientific evidence that supports the tool's health claims (e.g., published research, RCTs)	[15,17,18,20,21,32,33,39,42,56,57]
**2.c. Visible popularity metrics**					
	Ratings and user satisfaction	TEACH-Apps - MARS - NICE ESF	12 (30%)	Users' perceived value through users' reviews and ratings (as a proxy for quality, usefulness, or acceptability and popularity)	[15,18–21,33,39,41,50,54–56]
**2.d. Social aspects**					
	Social influence and endorsement	EUNetHTA - LCDH	7 (18%)	The possibilities for peer support, social networking, information sharing, and endorsement by HCPs	[20,31,38,42,44,54,60]
**3. Organizational assessment criteria**					
**3.a. Scalability and sustainability**					
	Cost effectiveness	ISAT - RE-AIM - APA - BIT - IOM - NICE ESF - EUNetHTA	16 (43%)	The balance between the costs and benefits arising from the tool's utilization. This refers to the tool's direct costs (purchase price, subscription, licensing…), but may also include costs associated with the tool's selection, staff training, setting up support mechanisms, and appropriate governance	[17,18,20,30,34,35,40,42,44,46,51,52,54,56,59,60]
	Maintenance	ISAT - RE-AIM - REP - TEACH-apps - BIT - CLIQ	13 (33%)	Commitment of the developers to maintaining their products in the long-term, by conducting periodic updates and maintenance (both from technical and content perspectives)	[18,20,30,34,35,37,42,48,51,53,54,59,60]
	Adoption and fidelity	ISAT - RE-AIM - BIT - NICE ESF - EUNetHTA - RACE	8 (20%)	Adoption, acceptability, and desirability of the tool, as well as its integration within clinical practice. System utilization and adherence to the tool	[20,30,34,35,56,57,59,60]
	Availability	EVALAPPS	2 (5%)	The guarantee of access to the tool and its data at any time, and its availability on the different operating systems (e.g., Android, iOS…)	[19,62]
**3.b. Healthcare organization**					
	Implementation	RE-AIM - TEACH-apps	4 (10%)	Assesses the extent to which the intervention was delivered as intended (e.g., feasibility of delivering all components of an intervention at a pre-determined date and time)	[18,34,35,59]

	Workforce and resources	ISAT - EUNetHTA	3 (8%)	the workforce required to scale-up the tool, and the implications for care process and care management	[30,59,60]
	Infrastructure	ISAT - EUNetHTA	3 (8%)	Assesses the readiness of the necessary infrastructure for the tool's implementation	[30,59,60]
**3.c. Health care context**					
	Strategic, political, and environmental contexts	ISAT - TACH-Apps - REP	3 (8%)	How favorable are the pre-conditions (strategic, political, and environmental contexts) that influence the scaling up of the eHealth tool. For example, the intervention's suitability to the socioeconomic context in question, considerations of foreign languages that the tool needs to support, literacy level, and the local regulatory environment	[18,30,60]
**3.d. Developer**					
	Transparency and credibility	APA - Pre-Cert - MedAd-AppQ	11 (28%)	Availability of information and credentials of the individuals and organizations involved in the development and funding of the tool	[15,16,18–20,39,41,42,48,51,53]
	Compliance and accountability	Pre-Cert - EUNetHTA	7 (18%)	Ethical conduct, clinical responsibility, and respecting the rules and regulations protecting patient's rights and societal interests (e.g., whether the tool was approved or certified by a regulatory body in the case of software as a medical device)	[15,19,20,51,53,59,60]
	Proactivity and interaction quality	Pre-Cert	2 (5%)	Interaction quality between the provider and the users, including responsiveness, after sales services, and customer orientation. Demonstration of excellence in a proactive approach to assessment of user needs, and continuous learning	[15,51]
	History of producing safe health products	Pre-Cert	1 (3%)	A history of producing safe and effective health care products	[15]
